# The impact of introducing an orthopaedic surgeon to an established multi-disciplinary foot ulcer clinic: a retrospective audit

**DOI:** 10.1186/1757-1146-4-S1-O41

**Published:** 2011-05-20

**Authors:** Erica Ryan, Joel Gurr, Cara Westphal

**Affiliations:** 1 Podiatry Department, Royal Perth Hospital, Perth, WA, 6000. Australia; 2 Podiatric Medicine Unit, School of Surgery, University of Western Australia, Nedlands, WA, 6009, Australia

## Background

The Multi-disciplinary Foot Ulcer Clinic (MDFUC) at Royal Perth Hospital (RPH) is an interdisciplinary clinical team that specialises in the management of patients with chronic foot ulceration and/or infection. The MDFUC was established in 2004 and is coordinated through the RPH Podiatry Department. In 2007, an Orthopaedic surgeon with foot and ankle expertise was added to the team. The aim of the audit was to review the impact of surgery on the healing of complex diabetic foot ulceration, to make informed comment on the value of orthopaedic surgery input into the MDFUC.

## Methods

A retrospective audit was conducted reviewing all cases that required orthopaedic foot surgery initiated through the MDFUC as a component of their management. Variables including patient demographics, surgery classification, types of procedures, pre and post surgery ulcer duration, complication rates and status one year post surgery were collected.

## Results

The audit identified 24 cases of orthopaedic surgery, of which preliminary analysis has been performed on 20 cases due to the availability of accurate data. 85% of the cases were complicated by osteomyelitis. The mean pre-surgical duration of the diabetic foot ulceration was 468 +/- 410 days. Post surgery, the mean healing time for the original ulcer was 61 +/- 44 days in the 19 (95%) cases where complete healing was achieved. All these cases remained healed at the 12 month follow-up. 2 cases superficially re-ulcerated at the original ulcer site within the 12 months but healed again after a short period and at least 2 cases developed superficial transfer lesions.

## Conclusion

The findings suggest that orthopaedic surgery is effective in promoting healing in selected cases of chronic and complex foot ulceration through the correction of underlying deformity, muscle imbalance and/or biomechanical anomaly. The benefits of surgery appear to be prolonged. The addition of an Orthopaedic surgeon to the MDFUC has been integral to the success of the clinic and added extra scope to the care available for patients. The results suggest that surgery should be considered much earlier in the management of chronic, non-healing diabetic foot ulceration.

